# Editorial: The role of hematopoietic and immune microenvironment in hematopoietic stem cell transplantation

**DOI:** 10.3389/fimmu.2023.1139193

**Published:** 2023-01-19

**Authors:** Xiaoqi Wang, Qifa Liu, Xi Zhang

**Affiliations:** ^1^ Medical Center of Hematology, Xinqiao Hospital. State Key Laboratory of Trauma, Burns and Combined Injury, Army Medical University, Chongqing, China; ^2^ Jinfeng Laboratory, Chongqing, China; ^3^ Department of Hematology, Nanfang Hospital, Southern Medical University, Guangzhou, China

**Keywords:** HSCT, hematopoietic microenvironment, immune microenvironment, poor graft function, GVHD

The hematopoietic microenvironment is a precise collection of cells and structures, including stromal cells, cytokines, and extracellular matrix, just like a niche. The biological interaction of these cells with the hematopoietic progenitor cells capable of reconstituting all hematopoietic lineages and differentiated progenitor cells and cells of each committed lineage has been intensively studied (1). The hematopoietic microenvironment is always compared to the “soil” demonstrating its strong and vital support for hematopoietic stem cell (HSC) physiological functions such as self-renewal, proliferation, differentiation, homing, migration, apoptosis, and aging (2, 3). The dysfunction of the hematopoietic microenvironment manifests primarily as a decrease in normal hematopoiesis and results in hematopoiesis failure. In addition, the subdued immune microenvironment would suppress the normal immune response to malignant cells, thereby impairing the immunity to leukemia, which contributes to the bone marrow microenvironment promoting malignant cell proliferation, chemoresistance, and relapse (4).

Allogeneic hematopoietic stem cell transplantation (allo-HSCT) is an important potentially curative therapeutic option for hematological malignancies. The growth of HSC and leukemia stem cells is also directly regulated by the bone marrow microenvironment; thus, the bone marrow microenvironment is one of the most important factors in HSCT success (5). However, complications such as transplantation failure (GF), graft versus host disease (GVHD) and recurrence are the main issues restricting the improvement of HSCT. To better treat hematological malignancies, it is necessary to enhance the safety and efficacy of HSCT and reduce transplantation-related complications and mortality. The key to achieving these goals is to remodel and repair the hematopoietic and immune microenvironment, with novel strategies in this field undergoing continuous development. Current disintegration concepts for targeted repair emphasize the development of both chemical drugs and cellular-based therapy. N-acetyl-L-cysteine (NAC) was reported to improve defective hematopoiesis by repairing dysfunctional bone marrow (BM) endothelial cells (EC) in BM failure diseases, such as poor graft function (PGF) or prolonged isolated thrombocytopenia (PT) after allo-HSCT *in vitro* study (6, 7), with the phase 3, open-label randomized trial performing to take in NAC to improve BM EC and reduce the incidence of PGF and PT from 22.5% to 7.5% (8). Eltrombopag, an oral thrombopoietin receptor agonist, was reported to be a safe and effective therapy for improving graft function (9). All-trans retinoic acid also represented a promising therapeutic approach in repairing hematopoietic environment impairment in patients with immune thrombocytopenia (10). Since the key to remodeling and repairing the BM microenvironment is the repair of the niche structure, cellular therapies such as MSC and EPC are able to maintain the stability of the BM microenvironment and regulate hematopoiesis and immunity. On one hand, MSC participates in the development of osteoblasts and the repair of the reticular matrix niche, thereby regulating the activity of HSC colonization and promoting hematopoietic recovery (11). During transplantation, MSC could expedite hematopoietic reconstitution by secreting chemokines that induce HSC to nest across blood vessels and into bone marrow (12, 13). It has also been demonstrated that MSC could stimulate residual hematopoietic tissue and treat various types of hematopoietic impairment (14). On the other hand, MSC combined with HSCT could restore immune function and reduce the incidence of GVHD (15). Our team previously demonstrated that MSC could regulate T-cell activation and reduce NK cell ratio to alleviate GVHD (16, 17), and MSC exerted immunosuppressive effects by predominantly chemokine receptor ligand (CXCL1-CXCR2) binding chemotactic myeloid-derived suppressor cells (MDSC) to GVHD target organs (18). In addition, the prospective phase 2 clinical trial conducted demonstrated that MSC were effective in reducing the incidence of GVHD by maintaining the immune state balance by upregulating the ratio of Th1, Treg, and memory B cells while simultaneously decreasing the ratio of Th2 and NK cells (19). Thus, cellular-based therapy can reshape the bone marrow microenvironment, restore hematopoietic ecology, and promote hematopoietic/immune recovery, which is more promising than the development of drug therapy.

This Frontiers in Immunology Research Topic presents the latest insights from preclinical studies and clinical studies of the hematopoietic and immune microenvironment in HSCT from two aspects, hematopoiesis and rejection, highlighting the underlying mechanisms and intervention strategies for reestablishing hematopoiesis and immune balance, shedding the light on improving the HSCT safety. The collection consists primarily of research articles and reviews addressing HSCT complications by repairing the hematopoietic and immune microenvironment to promote hematopoietic reconstruction and correct immune disorders, with an emphasis on clinical relevance and translational potential. Since PGF and thrombocytopenia are common complications associated with the poor hematopoietic reconstruction, the perspective article by Lin et al. provides an instructive overview of the secondary poor graft function (sPGF). They retrospectively reviewed 423 consecutive aplastic anemia (AA) patients to elucidate the incidence, clinical outcomes and risk factors of sPGF in haploidentical (haplo-) HSCT for AA patients, highlighting the pivotal insights afforded by clinical data, supporting evidence to sPGF prophylaxis for AA patients after haplo-HSCT. In addition, Zhu et al. conducts a retrospective study to evaluate the efficacy and safety of avatrombopag combined with MSC in patients with thrombocytopenia after allo-HSCT. The results indicated that combination of avatrombopag with MSC could promote platelet recovery and improve the survival rate, thus providing an optional strategy for the thrombocytopenia treatment.

In this collection, immune disorders mainly refers to the GVHD, another significant complication after HSCT. Meanwhile, the therapeutic effect of HSCT, mediated by alloimmunity towards malignant cells, is graft-versus-leukemia (GVL) activity mediated by alloreactive T cells that could eliminate residual disease and prevent relapse. Prevention of GVHD while preserving GVL activity remains a long-sought and elusive goal. Song et al. review the field and discuss recent advances in preclinical studies and clinical trials that have tested this compartmental approach for preventing GVHD while preserving GVL activity, providing novel insights for the translational potential of targeting this separation. Specifically, they focus on the approaches for tolerating infiltrating T cells within the GVHD target tissues through interaction between host tissue expression of PD-L1 and T cell expression of PD-1, thus preventing GVHD while allowing full activation and expansion of alloreactive T cells within lymphohematopoietic tissues so that the GVL activity remains intact. In addition, Gao et al. investigated the impact of Daratumumab (Dara), a humanized monoclonal antibody targeting the CD38, on xenogeneic GVHD (xeno-GVHD) model and GVL effects in a humanized murine model of transplantation. Their results demonstrated that Dara efficaciously mitigated GVHD through inhibiting CD8+ cytotoxic T cells proliferation, activation and differentiation, reducing expression of cytotoxic effector molecules, inflammatory cytokines and promoting regulatory T cells function, meanwhile Dara preserved GVL effect by the induction of Th17, Th1/17and Tc1/17 cells. Epigenetic agents, mainly including hypomethylating agents (HMAs) and histone deacetylase inhibitors (HDACi), have been regarded as candidates to mitigate GVHD without influencing GVL. Yang et al. review the application of epigenetic agents in the bridging treatment, preconditioning regimens, maintenance, preemptive and salvage therapy after HSCT focusing on the important insights garnered from these studies. Finally, since EPC have been of great interest to recover hematopoietic microenvironment after HSCT, Wang et al. estimate that EPC infusion could mobilize to damaged endothelium to reduce T cell infiltration and pathological endothelial activation, thereby mitigating endothelial damage. The authors determined its utility in the context of HSCT and posit the possibility of a broader application.

In summary, hematopoietic and immune microenvironments have proven to be increasingly significant for advancement of our understanding of improving HSCT safety and overcoming complications that occur following HSCT (20). Recent study has combined single-cell RNA-sequencing (scRNAseq) to investigate the precise timepoint at which HSC seeding occurs in the fetal BM and how the interactions occur between HSC and niche cells contributed to the hematopoiesis (21). As evidenced in this compilation, BM microenvironment structure repair and functional recovery have also led to the discovery of new scientific knowledge, allowed established concepts to be challenged and emerging concepts to be explored, facilitated the development of new therapeutics, and provided crucial insights that have guided clinical studies. With the development of new ideas, technologies, and tools, optimizing repairing strategy and deepening mechanisms studies are always on the way to empower academic research and translation into the clinic.

## Author contributions

All authors listed have made a substantial, direct, and intellectual contribution to the work and approved it for publication.
